# Monoclonal Gammopathies and the Bone Marrow Microenvironment: From Bench to Bedside and Then Back Again

**DOI:** 10.3390/hematolrep15010004

**Published:** 2023-01-09

**Authors:** Federica Plano, Anna Maria Corsale, Emilia Gigliotta, Giulia Camarda, Candida Vullo, Marta Di Simone, Mojtaba Shekarkar Azgomi, Maria Speciale, Melania Carlisi, Nadia Caccamo, Francesco Dieli, Serena Meraviglia, Sergio Siragusa, Cirino Botta

**Affiliations:** 1 Department of Health Promotion, Mother and Child Care, Internal Medicine and Medical Specialties, University of Palermo, 90127 Palermo, Italy; 2 Department of Biomedicine, Neurosciences and Advanced Diagnosis, University of Palermo, 90127 Palermo, Italy

**Keywords:** multiple myeloma, smoldering myeloma, monoclonal gammopathy of undetermined significance, bone marrow microenvironment, tumor associated immune cells

## Abstract

Multiple myeloma (MM) is an incurable hematologic malignancy characterized by a multistep evolutionary pathway, with an initial phase called monoclonal gammopathy of undetermined significance (MGUS), potentially evolving into the symptomatic disease, often preceded by an intermediate phase called “smoldering” MM (sMM). From a biological point of view, genomic alterations (translocations/deletions/mutations) are already present at the MGUS phase, thus rendering their role in disease evolution questionable. On the other hand, we currently know that changes in the bone marrow microenvironment (TME) could play a key role in MM evolution through a progressive shift towards a pro-inflammatory and immunosuppressive shape, which may drive cancer progression as well as clonal plasma cells migration, proliferation, survival, and drug resistance. Along this line, the major advancement in MM patients’ survival has been achieved by the introduction of microenvironment-oriented drugs (including immunomodulatory drugs and monoclonal antibodies). In this review, we summarized the role of the different components of the TME in MM evolution from MGUS as well as potential novel therapeutic targets/opportunities.

## 1. Introduction

Multiple Myeloma (MM) is a malignant disease characterized by proliferation of clonal plasma cells in the bone marrow, typically associated with organ damage due to the expansion of malignant cells or to the production of the monoclonal paraprotein. It is the second most common hematologic malignancy and the median age at diagnosis is 69 years, but 75% of patients receive a diagnosis above the age of 55 years [1,2]. While a specific etiology for MM could not be identified, age, gender, ethnicity, genetic predisposition, lifestyle, and environmental factors play a pivotal role.

MM can be considered a prototype for multi-step cancers; in fact, it is consistently preceded by two asymptomatic phases known as monoclonal gammopathy of undetermined significance (MGUS) and smoldering MM (SMM). Unfortunately, many cases of multiple myeloma often arise de novo because of the absence of signs or symptoms that characterize MGUS or SMM. Recent studies have shown how MGUS can be found in about 5% of the population above the age of 40 [3], with a prevalence increasing with age and a rate of progression from MGUS to MM of approximately 1% of patients per year [4]. On the other hand, the probability of evolution from SMM to MM decreases over time [5].

The clinical picture of MM is characterized by bone pain, kidney damage, anemia-related asthenia, and infection. The symptoms and various clinical subtypes of monoclonal gammopathies are described in detail in Table 1. 

MM is still considered a treatable but incurable disease. However, the framework of the life and care perspectives of MM patients have drastically changed in the last two decades, thanks to the introduction of new diagnostic tools and innovative therapeutic agents. 

From a biologic point of view, clonal plasma cells (cPCs) exhibit key genomic features (such as chromosomal aberrations) that are present in both active MM as well as in MGUS and SMM; however, only 1% of MGUS and 10% of SMM per year eventually evolve to overt MM, supporting the idea that genomic alterations are not sufficient, alone, for disease progression. Therefore, it is important to study epigenetic/phenotypic changes in several “normal” cells within the MM bone marrow (BM) microenvironment (TME) to fully understand the pathogenesis of MM and to improve its management and treatment [8].

Indeed, TME is characterized by a wide range of components such as hematopoietic stem cells, progenitor cells, endothelial cells, immune cells, mesenchymal stromal cells, osteoblasts, osteoclasts, adipocytes, extracellular matrix proteins, and growth factors that play an important role in supporting MM disease on multiple levels, including MM progression [9].

In this review, we analyze the central role of the bone marrow microenvironment in the multistep model of evolution from MGUS and SMM to MM and the potential therapeutic implications/opportunities.

## 2. Multiple Myeloma and Other Monoclonal Gammopathies: A Multistep Disease

### Genomic Aspects

Current experimental data support the idea that MM results from a neoplastic transformation that occurs in B cells of the post-germinative center, that is, in the terminal stages of B cell maturation and differentiation, most likely involving a memory B cell or a plasmablast. 

Genetic alterations involved in the pathogenesis of MM involve cytogenetic aberrations are often related to the translocations of the immunoglobulin heavy chain (IGH) locus on 14q32 with different partners (often oncogenes) and could be found in 40–50% of patients with monoclonal gammopathies [10]. These translocations mainly involve five chromosomal loci 11q13, 6p21, 4p16, 16q23, and 20q11 that contain the CCND1, CCND3, FGFR3/NSD2, MAF, and MAFB oncogenes, respectively. They lead to the overexpression of the oncogene juxtaposed to the 3′ intronic IGH enhancer. In particular, translocations t(11;14) and t(6;14) juxtapose the IGH enhancer with *CCND1* (15–20%) and *CCND3* (1–4%), respectively [11,12]. The cyclin D dysregulation induced by both translocations inactivates RB1 (retinoblastoma), allowing cell-cycle progression [12].

Translocations involving *maf* genes have been found in a minority of myeloma and these derive from IGH rearrangements with a locus in chromosome 16, most commonly t(14;16)(q32;q23) [13]. Further, studies have also suggested that the loss of chromosome 16 and/or increased expression of the FOPNL gene at 16p13 may be linked to poorer outcomes in myeloma [14]. 

These cytogenetic lesions are essential for the development of gammopathy, while a second event (“second hit”) is needed for neoplastic progression. These secondary lesions include the loss of chromosome 13, activating mutations in the NRAS and KRAS oncogenes, inactivating mutations or deletions of p53, and the inactivation of PTEN [15,16]. 

In the literature, many studies observed multiple significant mutations in the same tumor sample, including mutations in oncogenes whose function might be expected to be redundant [17]. For example, some patients had mutations in two of three oncogenes (NRAS, BRAF, and KRAS) or two mutations in KRAS, despite the fact that these mutations similarly activate the MAP kinase pathway. Both RAS mutations are associated with poor prognosis, aggressive disease phenotype and lower survival rates [18]. KRAS and NRAS mutations also promote the progression of MGUS in MM, and these oncogenes are mutated in up to 40% of newly diagnosed MM cases [19].

**Figure 1:**
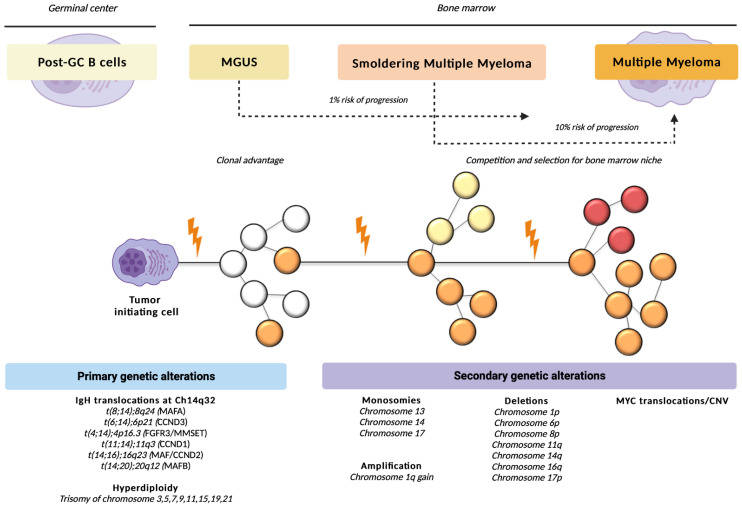
Genetic alterations involved in the pathogenesis of monoclonal gammopathies. Longitudinal evolution of cPCs according to current knowledge [13]. For the malignant transformation of a post-GC B cell to an MM cell, a genetic event is necessary, initiating the transition to the phase of MGUS. Malignant plasma cell accumulates new genetic mutations over time, acquiring growth advantage in a subclone and leading to further expansion of some clones (orange/red) and to the extinction of others (white, yellow). Abbreviations: cPCs, clonal plasma cells; post-GC, post-germinal center; MM, multiple myeloma; MGUS, monoclonal gammopathy of undetermined significance.

The secondary lesions mainly also affect MYC (8q24), whose rearrangements have recently been recognized as an independent negative prognostic factor in newly diagnosed MM patients [19]. It is uncommon in MGUS patients, but is present in 15% of SMM cases and 50% of cases of advanced disease [18] Figure 1.

Evidence that the mutations described above are already present in the early stages of the disease calls into question the process of linear tumorigenesis, which is characterized by the progressive acquisition of different mutations that confer a selective advantage to the neoplastic clone. 

In fact, according to the new theory of clonal evolution, genotypically different clones of plasma cells would coexist within the same patient [20]. Comparing the genetic characteristics of cells involved at different stages of the natural history of the disease, it was found that in about half of the cases the clone evidenced at relapse is genetically different from the clone present at diagnosis. Clonal evolution would therefore no longer be linear and involving a single clone of neoplastic cells, but branched and involving multiple clones whose balance depends on the ability of one of them to take over as a consequence of genetic instability and the acquisition of genetic abnormalities favorable to it [21].

In monoclonal gammopathies, sequencing and gene expression profiling studies have also identified numerous epigenetic defects, including the locus-specific DNA hypermethylation of cancer-related and B cell specific genes, genome-wide DNA hypomethylation and genetic defects, copy number variations, and/or abnormal expression patterns of various chromatin modifying enzymes. Importantly, these so-called epimutations contribute to genomic instability, disease progression, and a worse outcome [22].

Cytogenetic alterations play an important role in the risk of progression from SMM to active myeloma. First, in 2018, the Mayo Clinic group proposed a progression risk model based on three factors: FLCr > 20, BMPC > 20%, and serum M protein > 20 g/L. This model has been called “20/20/20” and considers patients with 0, 1, or ≥2 risk factors at low, intermediate, and high risk of progression, respectively [7]. Then, however, the International Myeloma Working Group (IMWG) recently conducted a multicenter retrospective study of patients with SMM to develop a robust risk stratification model in which to include high-risk cytogenetic abnormalities as a fourth risk factor in this model to further improve its accuracy [23]. This modified version of the 20/20/20 model is based on the evidence that certain cytogenetic abnormalities (t(4;14) translocation, t(14;16), gain of 1q, del13q, and monosomy 13) are an independent risk factor for progression [24]. Based on these new risk factors for progression, it has also been proposed that patients with high-risk SMM should be treated with survival benefits [16]. There is therefore a growing interest in identifying high-risk patients to explore treatment options in this specific subgroup. To understand the factors that determine an increased risk of disease progression and refractoriness to existing therapies, it is necessary to study not only the role of cytogenetic and molecular alterations, but also the complex interactions between plasma cells and cells of the bone marrow microenvironment.

## 3. The Cellular and Humoral Compartment of the Bone Marrow Niche

The BM niche appears to play an important role in the differentiation, migration, proliferation, survival, and drug resistance of cPCs [25]. The complexity of these processes is further supported by the bidirectional network between the TME and cPCs; indeed, the latter could interact with the microenvironment and modify its structure to favor the selection and expansion of subclones with precise phenotypic features [26]. 

In Table 2, we want to describe the cellular components of the bone marrow microenvironment.

**Figure 2:**
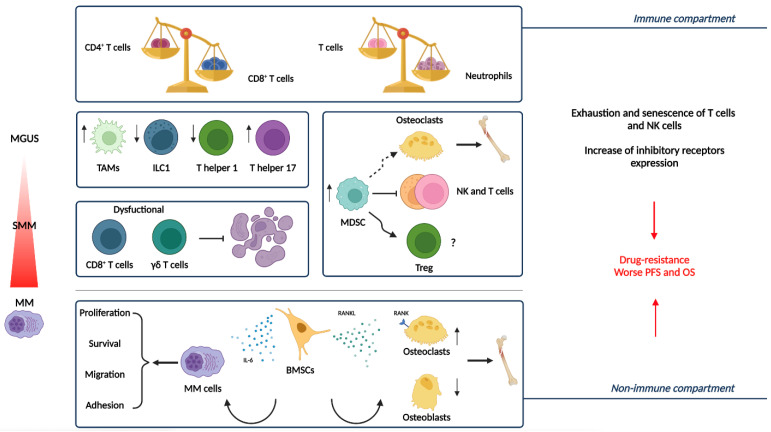
The immune and non-immune compartment of the Bone Marrow Niche in the progression from MGUS and SMM to active MM [25]. Representation of the main actors of the BM niche and the modification they undergo during disease evolution [26]. MM is characterized by quantitative and functional abnormalities related to immune cells such as inverted the CD4^+^/CD8^+^ T cell and T cells/neutrophils ratio. TAMs more dramatically increase in patients with advanced MM than in those with MGUS, supporting tumor cell proliferation and drug resistance. Moreover, as MM progresses, Th1 and ILC1 decrease, while Th17 increase. Due to the inhibition of T and NK cell activation and effector functions, as well as the promotion of Treg development or their differentiation into osteoclasts, MDSCs play a role in the progression of MM by obstructing the anti-tumor immune response and causing osteolytic lesions. Dysfunctional effector functions of cytotoxic T cells (e.g., CD8^+^ T cells and γδ T cells) contribute to impaired anti-tumor immune response. Among non-immune cells, BMSCs play a critical role in MM pathogenesis by cell contact, secretion of cytokines, growth factors, and extracellular vesicles. Osteoclast precursors differentiate into bone-resorbing osteoclasts due to the interaction between RANKL, expressed by BMSCs and upregulated during MM progression, and its receptor RANK on osteoclasts. BMSCs secrete cytokines, such as IL-6, that promote the expression of survival proteins, inducing the growth and survival of MM cells. These alterations, associated with exhaustion/senescence, and the increased expression of inhibitory receptors, cooperate to tumor growth, drug resistance, and immune escape in the context of MM. Abbreviations: MM, multiple myeloma; MGUS, monoclonal gammopathy of undetermined significance; TAMs, tumor-associated macrophages; MDSC, myeloid-derived suppressor cells; Th1, T helper 1; ILC1, innate lymphoid cells 1; Th17, T helper 17; Treg, T regulatory cells; BMSCs, bone marrow stromal cells; RANK, receptor activator of nuclear factor-κB.

The BM niche is composed of two large cell components, often referred to as immune and non-immune compartment or hematopoietic and non-hematopoietic cells, which together with the non-cellular counterparts cooperate for the maintenance, retention, and proliferation of HSC pools and their cells descendants [46,47,48] (Figure 2). 

In the next sections we discuss in detail the role of the different players.

### 3.1. Non-Immune Compartment

#### 3.1.1. Stromal Cells

BM stromal cells (BMSCs), also known as marrow mesenchymal stem cells (MSCs), are part of a non-hematopoietic multipotent cell pool able to differentiate into osteocytes, chondrocytes, adipocytes, tenocytes, as well as myocytes, neurons, and hepatocytes [49]. Although they represent a small part in the BM (only 0.01 to 0.001% of mononuclear cells) [50], they play a crucial role in supporting the upkeep and differentiation of hematopoietic lineages, regulating bone homeostasis, and contributing to the spatial delimitation of cellular niches [35,51]. Unfortunately, in pathological conditions, BMSCs, together with the components of the extracellular matrix, are essential for the generation of malignant niches, by releasing cytokines and growth factors and remodeling cell–cell contacts, making it favorable to the growth and survival of cPCs [34]. Specifically, MM cells interact with stromal cells through adhesion molecules such as VLA-4, LFA-1, MUC-1, or CD40: this cross-talk activates several pathways that induce cell cycle progression and inhibits pro-apoptotic signaling [52,53,54,55]. Compared with their normal counterparts, MM-BMSCs are characterized by increased cell size, reduced proliferation rate, and characteristic expression of senescence markers, together with the higher secretion of angiogenic factors [36,56]. Additionally, MM-BMSCs overexpress cytokines, which typically negatively affect osteogenic function [57,58,59], and reduce matrix mineralization and alkaline phosphatase activity [60]. It is also of note that transcriptomic analysis of MM-associated BMSCs highlighted clear differences with their non-myeloma counterparts, suggesting undergoing epigenetic modifications, that cause long-term phenotypic changes potentially associated with MM evolution [61,62].

#### 3.1.2. Bone Remodeling: Osteoblasts, Osteoclasts and Osteocytes

Other cell populations influencing MM evolution include osteoclasts (OCLs) and osteoblasts (OBLs). OCLs, derived from monocyte-macrophage lineage, are mainly responsible for bone resorption while OBLs, originating from MSCs, play a central role in bone formation. Physiologically, these cells interact with each other to maintain mineral homeostasis in a fine-tuned balance, strongly impaired in the presence of MM cells [38]. In fact, osteolytic lesions represent a major hallmark of multiple myeloma, which is almost completely absent in precursor conditions. This impairment of bone remodeling occurs due to an increase in pro-osteoclastic and anti-osteoblastic soluble factors, but also due to the direct cell–cell contacts established between MM cells and osteoclasts themselves [37,63,64]. 

The Receptor Activator of Nuclear Factor κ B (RANK) pathway is involved in this process. RANKL, expressed by OBLs, BMSCs and Th17, binds to its receptor RANK on osteoclast precursors and promotes their differentiation into bone-resorbing osteoclasts. This process is finely regulated by osteoprotegerin (OPG), a decoy receptor for RANKL released by osteoblasts, blocking osteoclast function and keeping the balance of bone formation and resorption. In the context of MM progression, RANKL is upregulated [65]. Particularly, MGUS patients showed higher RANK/OPG ratios than controls, but lower than MM patients. Thus, this highlights an early involvement of this pathway and an increase in osteoclastogenesis in early stages of disease, even in the absence of clinically relevant bone disease [66].

Bone lesions are identified by low-intensity total-body CT (WBLD-CT), PET-CT, and MRI. WBLD-CT allows comprehensive skeletal assessment in the search for osteolytic lesions and has the potential to clinically significantly change the definition that is assigned to myeloma at diagnosis, i.e., the difference between smoldering and active [67].

MRI plays a key role in staging the patient with SMM for proper clinical definition, especially where there is a negative conventional radiographic examination for bone involvement; as well as in staging patients with solitary bone plasmacytoma. In addition, MRI is a key examination both in discriminating between osteoporotic-based or myeloma-related vertebral failures and in accurately describing cord or nerve root compression, an essential implication for a possible surgical approach [68].

PET-CT is an instrumental examination that combines the identification of bone lesions by CT with a functional assessment of the metabolic activity of tumor cells [69].

A recent study has shown how the microenvironment could influence the activity of osteoblasts and osteoclasts through exosomes, which are often involved in generating a niche conducive to tumor growth in many tumors. Moreover, it is also known that the Notch3 signaling pathway is very important for MM cells’ growth and proliferation, and this pathway is upregulated upon binding to osteocytes [70]. This field is currently under active investigation and the understanding of changes in the role of the bone remodeling compartment during disease evolution will be of utmost importance for the design of novel agents able to prevent the instauration of bone lytic lesions.

### 3.2. The Immune Compartment

#### 3.2.1. Myeloid Cells

Several studies describe infiltrating tumor-associated macrophages (TAMs), which resemble M2-polarized macrophages, as important players in MM onset and progression, supporting tumor cell proliferation and resistance to drug-induced apoptosis. TAMs frequency dramatically increase in patients with advanced MM as compared with patients in partial/complete remission or subjects with MGUS, and associate to worse progression-free (PFS) and overall survival (OS) [9,71,72,73,74,75,76,77,78,79]. Indeed, a single-cell RNA sequencing study showed that mature CD14^+^ monocytes/macrophages showed defective antigen presentation due to the loss of HLA-II molecules, resulting in T cell suppression, already evident as early as the pre-neoplastic MGUS stage [80]. Moreover, myeloid-derived suppressor cells (MDSCs), a heterogeneous subset of immature myeloid cells, are involved in MM progression and treatment resistance with bidirectional interaction with myeloma cells within the TME [81,82,83,84,85]. Indeed, monocytic MDSCs (CD11b^+^ CD33^+^ CD15- CD14^+^ HLA-DR low/−) and polymorphonuclear MDSCs (CD11b^+^ CD33^+^ CD15^+^ CD14- HLA-DR low/−) progressively increased from pre-neoplastic conditions through MM at diagnosis to relapse and correlated with poor OS [86]. MDSCs hampered the anti-tumor immune response by multiple mechanisms dependent on direct cell-to-cell contact or exosome intercellular communication [87,88,89] The MDSC-mediated induction of the immunosuppressive milieu in MM was strictly dependent on the inhibition of T and NK cells activation and effector functions as well as by inducing Treg development [90] or differencing themselves into osteoclasts, contributing to the formation of osteolytic lesions [91]. Interestingly, in MM patients, CD11b^+^CD13^+^CD16^+^ mature neutrophils should be considered the true PMN-MDSCs [92]. Neutrophils have also been involved in MM progression and are significantly different among healthy, MGUS, and MM subjects [93]. They may support the increased susceptibility to infection and the impaired anti-tumor immune responses due to defective phagocytosis and oxidative burst [94]. Furthermore, only mature neutrophils influenced the patient outcome in newly diagnosed MM patients [92]; indeed, a high mature neutrophil/T-cell ratio was associated with reduced PFS [92,95,96]. The presence of mature neutrophils decreased T-cell proliferation and, when depleted, the cytotoxic functions of T cells increased, engaged by a BCMA × CD3-bispecific antibody [92]. The expression of some genes (e.g., CSK, GSA, MEGF, PGM1, and PROK2), associated with the progression from MGUS through active MM, have been upregulated in high-density neutrophils of these patients [94].

During MGUS to MM progression, the phenotypical and functional alterations of dendritic cells (DC) have also been described. In MM patients, a 50% reduction of myeloid DCs (BDCA1^+^) and plasmacytoid DCs (pDCs) (BDCA2^+^) within PBMCs was observed [97,98,99], independently of disease stage, compared to healthy controls. Instead, they have been demonstrated to accumulate in the BM of MM patients as compared to those with MGUS (4–5), supporting the proliferation of cPCs [99,100,101]. Additionally, they have been demonstrated to promote Th17 differentiation and the generation of a pro-inflammatory TME which is prone to the development of lytic bone lesions [102]. Peripheral myeloid DCs and pDCs in MM patients were also characterized by the downregulated expression of CCR5, CCR7, DEC-205, HLA-DR, and co-stimulatory molecules, and a defective IFN-γ production, associated with impaired T cell [97,98] proliferation and activation which impair their migration and antigen-uptake capability. It is worth nothing that, consistent with the progressive accumulation of DCs in the BM, a progressive upregulation of CD28 expression, a receptor for CD80/CD86, was described on tumor plasma cells during the MGUS-to-MM transition. Following CD28-mediated interaction between plasma cells and BM myeloid DCs, the expression of proteasome subunits was downregulated in these cells, favoring their escape from CD8^+^ T-cell killing. To be noted that some studies described a normal numbers of DCs in MM patients [44,103].

#### 3.2.2. Lymphoid Cells

T lymphocyte subsets play an active role in tumor immunosurveillance in MM and their quantitative and functional abnormalities have been identified, beginning at the MGUS stage [104,105]. Compared to MGUS/SMM patients or healthy individuals, BMMCs and PBMCs of MM patients were characterized by an inverted ratio of CD4^+^:CD8^+^ T cells associated with lower PFS and OS, as well as a higher relapsing probability [80,104,106,107,108]. Interestingly, CD4^+^ and CD8^+^ T cells were able to mount an intensive response against autologous premalignant cells in MGUS patients but not in those with MM, suggesting that these cells are functionally compromised [109,110]. On the one hand, this could be explained by the fact that T lymphocytes from MM patients were functionally exhausted/senescent and have a significantly higher expression of inhibitory receptors than those with MGUS/SMM or healthy subjects [111,112,113]. The cytotoxic activity of CD8^+^ T cells could be inhibited by the alterations in the antigen processing-presenting machinery of transformed plasma cells [110]. Patients with MM were also characterized by a depletion of memory CD8^+^ T cells [80] and a skewed Treg/Th17 ratio [114,115,116,117,118] compared to MGUS patients, indicating a more suppressive environment and associated with worse OS [84]. The presence of Th17 cells in the BM from MM patients correlated with clinicopathological characteristics [119] and lytic bone disease development [117]. 

The role of Tregs is still a matter of debate and there are conflicting reports about their [116,120,121,122] increased [120,121], decreased or unchanged frequency [123,124], and correlation with survival parameters [125]. 

In fact, increased numbers of Tregs in bone marrow have been shown to correlate with adverse clinical features, such as hypercalcemia, decreased normal plasma cell counts, and IgA myeloma subtype [104,124]. 

The frequency’s discrepancy may be likely explained by the heterogeneity of samples that have been studied (i.e., whole-blood compartment, peripheral-blood mononuclear cells, bone marrow) and the variety of gating strategies of Tregs which may lead to different results regarding Tregs frequencies in MM patients [126]. There is also no consistency on how Tregs numbers are reported (either % frequencies or absolute values).

CTLA4^+^ or PD1^+^ Tregs were increased in the BM from MM patients compared to those of MGUS/SMM or healthy donors [108,127]. 

In addition, a senescent Treg cell subset with partial suppressive function, identified as CD28-CD4^+^FoxP3^+^, was significantly higher in PB and BM in MM patients than those with MGUS [116,123,128]. It is therefore conceivable that most of the positive or negative activity of these cells depends upon the microenvironment in which they are included: it is clear that for patients whose MM depends on an over-inflamed microenvironment (especially if Th17-dependent), an increase in Tregs infiltration could be beneficial, while, on the other hands, for non-inflamed tumors, the excess of Tregs could contribute to the immune-exclusion.

Another one of the earliest cell subsets enriched in the TME during MM evolution is innate lymphoid cells (ILCs), which could be detected as early as pre-neoplastic conditions [129]. MGUS patients are characterized by increased bone marrow ILCs MGUS patients are characterized by increased bone marrow ILCs showing a preva-lence of IFN-γ–producing group 1 ILCs (declining in asymptomatic MM patients) and a reduction of IL-13 producing group 2 ILCs [130]. 

In particular, NK cells, the prototypic member of group 1 ILCs, decreased their frequency in MM patients in advanced disease stages with poor prognosis compared to controls and those with MGUS [131,132,133,134,135]. Due to a reduced expression of activating receptors and their ligands (e.g., CD16, NCR3/NKp30, NKG2D, CD244/2B4/p38 and DNAM-1) [136,137,138], and increased expression of the inhibitory receptors such as KIR2DL1, PD1, TIM3 and TIGIT [139,140] by the surrounding TME, NK cell–mediated killing of cPCs was impaired in MM patients. In light of this, restoring or enhancing the effector functions of NK cells has been one of the recent immunotherapeutic approaches for the treatment of MM [141].

Instead, there are no significant differences in γδ T cell counts between MGUS and MM patients [142], but their accumulation was described at the SMM stage [80,143,144,145,146,147,148,149,150,151,152,153].

### 3.3. Soluble Factors Promoting Tumor Evolution

Apart from the cellular compartment, the BM niche is even composed by soluble factors, such as cytokines and growth factors, and physical interactions with stromal cells and extracellular matrix (ECM) molecules, all potentially involved in myeloma evolution. 

Both normal and malignant plasma cells can reach and colonize BM using the sinusoids as an entry route [34]. The main molecule mediating homing, lodging, and retention of those cells into the BM is the chemokine receptor CXCR4 [154,155]. Two other essential adhesion molecules for MM cells are CD49d and CD44, which contribute to the MM cells’ trafficking to the final destination [156,157]. In contrast to normal plasma cells, cPCs have higher expression levels of cell adhesion molecules (CAM) such as VLA-4, N-CAM (CD56), CXCR4, and MAC-1. Among others, CXCR4 is a promising target for impairing MM cell trafficking, and new agents are currently under investigation [158,159]. Once reaching the BM niche, cPCs should re-educate the microenvironment. Chemokine and cytokine reprogramming in order to maintain a favorable microenvironment represents the first step in this direction [160]. Interestingly, monitoring these modulations could serve as predictive markers for disease progression/evolution. For instance, an aberrant expression of CCL2 and CCL3 is involved in chemoresistance development and correlates with the disease stage [161]. In addition, both chemokines affect the macrophages’ infiltration and polarization into TAM in BM [162]. Along the same lines, another soluble factor, IL-32, has been linked with worse survival and a more advanced clinical stage of MM [163,164]. Indeed IL-32α induces IL-6 production in BM stromal cells which in turn promotes MM cell growth and prevents apoptosis through JAK/STAT and RAS/MAPKs pathway activation [165,166]. 

Angiogenesis increases progressively along the spectrum of plasma cell disorders from MGUS to smoldering MM to MM [122]. This phenomenon is further supported by the overexpression of vascular endothelial growth factor (VEGF), hepatocyte growth factor, and basic fibroblast growth in MM cell lines [167] as well as by the increase in the surrounding notch signaling network between MM cells, bone marrow cells, and endothelial cells [168]. In summary, enhancing homing chemokines, pro-inflammatory factors as well as angiogenesis provides a suitable niche for supporting the tumor growth and clinical evolution of monoclonal gammopathies, thus providing the rationale for potential future therapeutic targeting. 

## 4. Bone Marrow Modulating Agents: Clinical Applications

MGUS and SMM represent useful models for studying multiple myeloma precursor disease as well as for developing early intervention strategies [169,170].

Currently, outside of clinical trials, the management of MGUS and SMM is represented by watch and wait until a myeloma-defining event (MDE) occurs (lack of benefit with old trials). Indeed, previous studies did not show a benefit in terms of reduction in progression and improvement of overall survival (OS). Both the study by Hjorth [171] and subsequent attempts by Riccardi [172,173] had shown, in fact, the ineffectiveness of using melphalan with prednisone in the treatment of MDE. Other attempts were made with pamidronate [174,175], or zoledronic acid [176]. In both cases, antiproliferative, proapoptotic, antiangiogenesis, and direct cytotoxicity effects were sought to be exploited [177]. However, compared with a reduction in the incidence of skeletal events, a positive impact in overall survival was not achieved.

Thanks to progress in the understanding of disease biology and introduction of newer therapies that can restore the immune system, with better efficacy and lower toxicity, it is possible to reach deeper responses and improve longer survival for patients with active MM. These advances [176] have also challenged the management of SMM, especially in high-risk patients, raising the question of whether earlier treatment could: (1) avoid or delay the progression to MM; (2) prevent the severe complications of end-organ damage; and (3) hopefully cure some SMM patients.

Here, we report the most promising therapy in this setting, while others, investigated with unclear results, are reported in Table 3 (e.g., siltuximab, ixazomib, pembrolizumab, ibrutinib).

Immunomodulatory drugs (IMiDs) mainly act by binding to CELEBRON, thus changing the conformation of the ubiquitination machinery and inducing the degradation of Ikaros and Aiolos transcription factors in both normal immune cells and malignant plasma cells.

In turn, the final result mainly include the suppression of VEGF gene and pro-apoptotic activity as well as the induction of several immune-activating cytokines such as IL2.

Two prospective phase III studies have provided results to support the use of lenalidomide (± dexamethasone) in high risk SMM patients. The pivotal Spanish QuiRedex phase III trial (NCT00480363) randomized 119 patients with high risk SMM to receive nine induction cycles with lenalidomide + dexamethasone followed by only observation or maintenance with lenalidomide alone for 2 years [178,179]. Updated results after a median follow-up of 10.8 years revealed a 46% reduction in the risk of death and 73% in the risk of progression for early treatment as compared to observation [24]. This study pointed to the fact that the immunomodulatory activity of lenalidomide induces a switch from a tolerogenic to an effector microenvironment thus reactivating the immune surveillance against the tumor cells of high-risk SMM patients [180]. This study suggests that early treatment with lenalidomide does not induce chemoresistant clones and early therapy in SMM does not negatively impact the following treatments.

The second trial, the ECOG E3A06 phase III trial (NCT01169337), assessed the efficacy of lenalidomide monotherapy compared with observation in intermediate/high-risk SMM patients [178,181]. Response to therapy was observed in 50% of patients in the lenalidomide arm. With a median follow-up of 35 months, PFS was significantly longer with lenalidomide than with observation, indicating a 72% decrease in the risk of progression, especially in high-risk SMM patients. It is of note that both studies include patients who today (due to the introduction of myeloma defining events) would have been classified as active myeloma, making the generalization of these results very difficult.

### 4.1. Carfilzomib, Lenalidomide, Desametasone

The activity of carfilzomib is based on the irreversible binding to proteasome complex which determine the induction of unfolded protein stress response [182].

In a US pilot study (NCT01572480), 18 high-risk SMM patients were treated with the carfilzomib, lenalidomide + dexamethasone (KRd) regimen resulting in an ORR of 100% [183]. After a median follow-up of 43.3 months, 63% of patients remained MRD-negative, with estimated 4-year PFS and OS rates of 71% and 100%, respectively. A subsequent phase II study in 52 high-risk SMM patients, assessed eight cycles of KRd followed by 2 years of lenalidomide maintenance (KRd-R) [184]. After a median follow-up of 27.3 months, the ORR was 100%; only 10% of patients had developed MM after 5 years.

The GEM-CESAR trial is a phase II, single-arm trial focusing on high/ultra-high-risk SMM patients [185]. After six induction cycles of KRd, followed by high-dose melphalan and autologous stem cell transplantation (ASCT) as an intensification therapy, they received two consolidation cycles with KRd and maintenance with Rd for up to 2 years. Updated results [186] indicate that the ORR was 98% after induction, 98% after ASCT, and 100% after consolidation; 68.6% of patients reached complete remission or better after consolidation, with 55% of them achieving MRD negativity.

Unfortunately, none of these trials has a control arm so that we still do not know if it is better to treat SMM patients early despite the cost of drug-related toxicities [187].

### 4.2. Zoledronate

Thanks to their specific characteristics, several studies evaluated the possibility to apply γδ T cells as candidates for immunotherapeutic approaches in hematological malignancies, including MM [143]. In vitro expanded γδ T cells by BrHPP, or zoledronate plus IL-2 or IL-15, exerted cytotoxicity against autologous MM cells as well as MM cell lines [144,145,146,147,148], but this effect was abrogated by γδ T cell depletion [149] or by the mevastatin-induced upstream blockade of the mevalonate pathway [150]. Moreover, the in vivo administration of zoledronate-activated Vγ9Vδ2 T cells proved to be a safe and promising immunotherapy strategy for the setting care of MM patients [151,152]. Unfortunately, the BM microenvironment in the context of MM is highly suppressive and makes BM Vγ9Vδ2 T cells more dysfunctional. Before and after zoledronate stimulation, BM Vγ9Vδ2 T cells of MM patients expressed high levels of immune checkpoint receptor PD-1, surrounded by PD-L1^+^ myeloma cells and increased numbers of PD-L1^+^ MDSC. The BM Vγ9Vδ2 T cell dysfunction was already detected in patients with MGUS as well as in those with MM in remission or relapse [153]. This suggests that Vγ9Vδ2 T cells, more than other immune effector cells, were hampered by a suppressive microenvironment in MM progression.

Interestingly, a study was conducted on treatment with IL2 and zoledronic acid as a maintenance therapy after autologous transplantation in patients with multiple myeloma [188]. However, these studies did not show a benefit compared with other standard maintenance therapies, such as lenalidomide, because the effect of IL2 and zoledronic acid on T lymphocytes is greatly impaired by the immunosuppressive bone marrow microenvironment in multiple myeloma.

### 4.3. Curcumin

Curcuma longa (turmeric) is a tropical plant native to southern and southeastern tropical Asia. The most active component in turmeric is curcumin [189]. In a select group of MGUS patients, oral curcumin at a dose of 4 g daily has been shown to:

- inhibit the proliferation and induce apoptosis in MM cells [190] through the downregulation of IL-6 and NF-kB;
- inhibit osteoclastogenesis [191] through the suppression of RANKL signaling;
- reduce bone turnover;
- decrease paraprotein load [192].

Between January and September 2010, 19 patients with MGUS and 17 patients with SMM [193] who were not receiving chemotherapy or bisphosphonates, were enlisted into a randomized, double-blind placebo-controlled study, followed by an open-label extension study using an 8 g dose to assess the effect of curcumin on FLC response and bone turnover [194]. Phase 1 clinical trials indicate tolerability and safety at doses as high as 8 g/day [195,196]. In this study, curcumin (4 and 8 g daily) decreased the free light-chain ratio (rFLC) (235 and 236%), the involved free light-chain (iFLC) (28 and 210%), and the difference between clonal and nonclonal light-chain (dFLC) (29 and 211%) in both MGUS and SMM patients. Significant reductions were also seen in total serum protein, serum creatinine levels and random urinary protein concentrations, but not in the serum paraprotein concentration. This is the first randomized study to show a potential beneficial effect of curcumin on FLC in MGUS and SMM patients, maybe due to a prolonged tumor suppressor effect, anti-inflammatory activity, immune-modulating and anti-angiogenetic effects.

Patients with an abnormal rFLC at baseline showed a greater response than patients with a normal rFLC. A decrease in rFLC was accompanied by a significant increase (at 8 g) in the uninvolved free light-chain.

In addition, patients were evaluated for tumor response every 8 weeks using computed tomography or magnetic resonance imaging, as well as monthly chest radiographs, and for changes in quality of life using the GLQ-C30 Quality of Life Questionnaire (version 2.0) before treatment and monthly during treatment [197]. Three significant changes in quality of life scores were recorded: one patient noted significant improvement after 1 month of treatment; two patients worsened after 2 months of treatment, both with radiologically progressive disease.

Interestingly, none of the 25 patients who completed the 4 g study (which includes the 18 on 8 g/day) have progressed to active disease 1 year after the study has been completed. These findings suggest that curcumin might have the potential to slow the disease process in patients with MGUS and SMM and that future studies should assess the role of curcumin in patients at risk of transformation.

## 5. Targeting MM Cells to Activate the Immune System: Monoclonal Antibodies and Vaccines

### 5.1. Daratumumab and Isatuximab (Anti-CD38)

Daratumumab binds CD38 surface antigen on malignant plasma cells induces antibody-dependent cellular cytotoxicity (ADCC), direct cytotoxicity, and bone remodeling. Daratumumab also inhibits the adhesion between myeloma cells and BMSCs, releasing cell adhesion-mediated drug resistance (CAM-DR), an important molecule for the adhesion of tumor cell integrins to stromal fibroblasts or to components of the extracellular matrix [198].

The randomized phase II CENTAURUS study (NCT02316106) evaluated daratumumab as a single agent in three different treatment schedules (extended intense, extended intermediate, or short dosing) in 123 intermediate/high-risk SMM patients [199]. After a median follow-up of 26 months, the complete remission rates were 4.9%, 9.8%, and 0%, respectively. Based on these data, the ongoing randomized phase III AQUILA study (NCT03301220) is investigating the long dosing schedule in 360 high-risk SMM patients, comparing subcutaneous daratumumab for up to 39 cycles versus watch and wait [200]. Preliminary results look promising, although adequate follow-up is needed to detect the potential benefit on OS [201].

Another ongoing phase III randomized trial (NCT03937635, DETER-SMM) is comparing lenalidomide + dexamethasone versus daratumumab, lenalidomide + dexamethasone (DRd) in 280 high-risk SMM patients.

The ongoing, phase II ASCENT trial (NCT03289299) is evaluating 12 cycles of a daratumumab, carfilzomib, lenalidomide and dexamethasone (DKRd) regimen, followed by 12 cycles of Daratumumab + lenalidomide maintenance, without autologous stem cell transplantation in high-risk SMM patients.

A single-arm phase II study (NCT03236428) is evaluating intravenous daratumumab for up to 20 cycles as single agent in 28 lower-risk SMM patients [23]. Partial response and at least very good partial response were achieved in 53% and 20%, respectively. No deaths, progression, or therapy discontinuations due to toxicity have occurred.

Isatuximab is a CD38-targeting immunoglobulin G1 monoclonal antibody that eliminates MM cells via antibody-dependent cellular cytotoxicity, antibody-dependent cellular phagocytosis, complement-dependent cytotoxicity, and direct apoptosis. It may also affect the tumor immunosuppressive environment via inhibition of CD38 adenosinergic activity [202,203].

Regarding Isatuximab, a phase II study (NCT02960555) is exploring the efficacy of intravenous isatuximab administered at decreasing intervals up to 30 weeks, in 24 high-risk SMM patients [204]. Five patients interrupted treatment, two because of progression to active MM. The best responses included partial remission (42%), very good partial remission (17%), and complete remission with MRD negativity (5%).

A phase III, randomized, multicenter study comparing isatuximab, lenalidomide + dexamethasone (IRd) versus lenalidomide + dexamethasone in higher-risk SMM within 5 years is about to start (NCT04270409) [24].

Overall, we still do not have any indication to support the use of anti-CD38 mAbs in SMM patients.

### 5.2. Vaccines

PVX-410 is a vaccine carrying a combination of four peptides, specifically targeting the highly overexpressed plasma cell antigens XBP1, CD138, and CS1/SLAMF7. A phase I/IIa multicenter, dose-escalation study (NCT01718899) enrolled 22 intermediate/high-risk SMM patients who received PVX410, with or without lenalidomide [205]. In the cohort receiving PVX-410 alone, 3 of 12 patients progressed, with a median time to progression of 36 weeks. In the combination cohort, 5 of 12 patients showed a clinical response, with one patient progressing and a median time to progression not reached. PVX-410 is also under investigation in a phase I trial (NCT02886065) in SMM in combination with the selective histone-deacetylase inhibitor citarinostat ± lenalidomide.

## 6. Conclusions

There is clear evidence that disease evolution from MGUS to SMM to active MM depends upon complex genomic alterations of plasma cells coupled with extensive reprogramming of the BOM microenvironment. We know, therefore, that the cause of multiple myeloma is a cytogenetic/molecular alteration affecting the plasma cell, but there is no single factor that alone with a cause-and-effect relationship that determines the occurrence of such mutations [206].

Unfortunately, the majority of the mechanisms underlying these evolutions are far to be elucidated and many efforts should still be made to identify the biomarkers of disease evolution as well as new therapeutic targets. The currently ongoing iMMunocell, IstopMM, and NOmoreMGUS studies are clear efforts in this direction, with preliminary results which are really promising. Along the same line, different trials are evaluating the impact of early treatments on disease evolution. Further help will be provided by the application of innovative imaging approaches (radiomic) to MGUS/SMM patients, aiming to identify biological correlates able to predict disease progression and evolution [207]. However, while all ongoing studies seem to identify a survival benefit, they do not include an untreated control group, thus no definitive conclusions can yet be reached. Still, more and deep knowledge is needed aiming to identify patient-specific immune-profile and to design, in a translational and personalized way, specific immunotherapies for MM precursor diseases, finally overcoming the controversial “to treat or not to treat” question.

## Figures and Tables

**Table 1:** Clinical subtypes of “premalignant” plasma cell disorders.

Plasma Cell Disorder	Diagnosis	Note
Non-IgM MGUS (IgG, IgA, IgD)	Serum monoclonal protein < 3 g/dL) Clonal plasma cells in the bone marrow < 10% * Absence of end-organ damage (CRAB symptoms) or amyloidosis that can be attributed to the plasma cell proliferative disorder	*Up to 85% of MGUS cases* *Annual risk of progression of 1%* * Bone marrow can be deferred in patients with low-risk MGUS (IgG type, M protein < 15 gm/L, normal free light chain ratio) in whom there are no clinical features concerning myeloma
IgM MGUS	Serum IgM monoclonal protein < 3 g/dL) Clonal plasma cells in the bone marrow < 10% No evidence of anemia, constitutional symptoms, hyperviscosity, lymphadenopathy, hepatosplenomegaly, or other end-organ damage that can be attributed to the plasma cell proliferative disorder	*15% of MGUS cases*
Light chain MGUS	Abnormal FCL ratio Increased level of involved FLC No immunoglobulin heavy chain expression on immunofissation Absence of CRAB symptoms or amyloidosis that can be attributed to the plasma cell proliferative disorder Clonal plasma cells in the bone marrow < 10% Urinary monoclonal protein < 500 mg/24 h	
Monoclonal gammopathy of renal significance (MGRS)	One or more renal lesions related to the monoclonal immunoglobulin produced The underlying B-cell or plasma cell clone neither causes tumor complications nor meets any current hematologic criteria for specific therapy The diagnosis of MGRS can only be established with renal biopsy	*Based on consensus report of the International Kidney and Monoclonal Gammopathy Research Group* [6]
Monoclonal gammopathy of neurologic significance (MGNS)	Chronic neuropathy with sensory ataxia, ocular, and/or bulbar motor weakness in the presence of a monoclonal IgM reacting against gangliosides containing disialosyl epitopes The diagnosis of MGNS is one of exclusion	
Smoldering Multiple Myeloma	Serum monoclonal protein (IgM or IgA) ≥ 3 g/dL) or urinary monoclonal protein ≥ 500 mg/24 h Clonal plasma cells in the bone marrow 10–60% Absence of MDE or amyloidosis	*Based on Mayo 2018 criteria* [7] *there are three groups of patients:* *High risk (47% risk of progression in the first 2 years)* *Intermediate risk (26% risk of progression in the first 2 years)* *Low risk (10% risk of progression in the first 2 years)*

Abbreviations: Ig, immunoglobulin; MGUS, monoclonal gammopathy of undetermined significance; CRAB symptoms, hypercalcemia, renal insufficiency, anemia, and bone lesions; MDE, myeloma defining events; FLC, free light chain.

**Table 2:** Cellular component of the bone marrow microenvironment in MM pathogenesis.

Cellular Component	Function	References
MDSCs	Hamper the anti-tumor immune response by multiple mechanisms dependent on: direct cell-to-cell contact. exosome intercellular communication promoting their expansion and activation through STAT-1 and STAT-3 pathways and increasing the release of nitric oxide, enhancing their suppressive activity on T cells. Inhibit T and NK cell activation and effector functions through several mechanisms: amino acid depletion (e.g., arginine and cysteine) and production of immunosuppressive metabolites (e.g., kynurenine). generation of reactive oxygen species, reactive nitrogen species, and peroxynitrite. immunosuppressive cytokine production, such as IL-6, IL-10, and IL-1β. direct engagement of T cell inhibitory and apoptotic receptors (e.g., PD-1, TIM-3, Fas). inhibition of naïve T cell homing to the lympho nodes due to cleavage of L-selectin/CD62L by disintegrin and ADAM17. production of extracellular adenosine and their accumulation and migration mediated by S100 calcium-binding protein A8/A9. anergy of NK cell is induced through membrane-bound TGF-β1. Promote tumor angiogenesis by MMP secretion or direct differentiation into endothelial cells. Induce Treg development Differentiate into osteoclasts, contributing to the formation of osteolytic lesions	[27,28,29,30,31,32]
BMSCs	Generate premetastatic niche: Release of cytokines and growth factors. Remodelling cell–cell contacts. Establish a crosstalk with MM cells: Increased expression of adhesion molecules (e.g., VLA-4, LFA-1, MUC-1, CD40). Induction of MM cells proliferation, inhibiting pro-apoptotic signaling pathways. Impair the osteogenic differentiation: Decreased expression of bone formation markers and transcriptional factors. Overexpression of cytokines that negatively affected osteogenic function.	[8,33,34,35,36]
Osteoclasts	Create an immunosuppressive microenvironment: Inhibition of activated CD4^+^ and CD8^+^ T cells proliferation. Increased expression and secretion of immune checkpoint molecules (e.g., PDL-1, galectin-9, APRIL, HVEM, CD200, IDO, CD38) resulting in T cell apoptosis. Degradation of bone matrix: Extra- and intra-proteolytic activity. Inhibition of bone marrow reabsorption, inhibiting osteoblasts differentiation. Higher differentiation due to: Hyperactivation of RANK-RANKL-OPG signaling. Overexpression of proinflammatory cytokines such as IL-6. Formation of osteolytic lesions	[37,38,39]
Dendritic cells	Immunosuppressive and tumor-promoting actions: Impaired antigen presentation capacities. Defective IFN-γ production. Promote Th17 differentiation and following IL-17 accumulation, indirectly favoring the osteoclastogenesis. Proteasome subunit downregulation decreases the expression of tumor antigen peptides on tumor plasma cells, enabling them to escape from CD8^+^ T cell recognition and killing using CD80/86/CD28 signaling. Promote tumor plasma cell growth, survival, and drug resistance. Express high levels of PD-L1.	[40,41]
TAMs	Support MM cells proliferation and survival through activation of the IL-6/JAK/STAT3 pathway. Decrease T cell proliferation and activation through the downregulation of IFN-γ, IL-2, and TNF-α secretion. Immune suppressive activity mediated by their secretion of IL-6, IL-10, activating Tregs and M2 macrophages, and TGF-β, inhibiting both cytotoxic T-cells and NK cells Angiogenic and vasculogenic activities: Angiogenic cytokines and proliferation markers positively correlate with IL-10 secretion. Promotion of HUVECs proliferation, migration, and tube formation in vitro. Neovascularization by vasculogenic mimicry and, indirectly, by secreting a broad range of proangiogenic factors (e.g., VEGF, IL-8, FGF-2, MMPs, COX-2, and CSF-1). Induce immune escape via the macrophage immune checkpoint SIRPα which binds CD47 overexpressed in CD138^+^ tumor cells.	[25,42]
Neutrophils	Overexpress IFN-γ, resulting in increased autophagy flux and JAK-2/STAT3 pathway activation, which supports their promotion of pro-inflammatory and survival signals within MM niches. Produce arginase that inhibits T cell activation and proliferation. Reduced lysozyme activity and increased secretion of the amino acid degrading enzyme.	[9,43]
T cells	Th17 cells: Induce myeloma cell growth and colony formation via IL-17 receptor. Inhibit Th1 immune response. Plays a role in osteoclast-mediated lytic bone disease. T-cell exhaustion associated with high expression of immune-checkpoint ligands on MM cells are responsible for the immune evasion.	[44,45]

Abbreviations: MDSCs, myeloid-derived suppressor cells; BMSCs, bone marrow stromal cells, NK, natural killer; ADAM17, metalloproteinase domain-containing protein 17; MM, multiple myeloma; TAMs, tumor-associated macrophages; VLA-4, integrins alpha-4/beta-1; LFA-1, lymphocyte function-associated antigen 1; MUC-1, mucin 1; RANK, receptor activator of nuclear factor-κB; RANKL, receptor activator of nuclear factor-κB ligand; OPG, osteoprotegerin; PD-L1, programmed death-ligand 1; APRIL, proliferation-inducing ligand; HVEM, herpesvirus entry mediator; IDO, indoleamine 2,3-dioxygenase; HUVECs, human umbilical vein endothelial cells; VEGF, vascular endothelial growth factor; FGF-2, fibroblast growth factor-2; MMPs, metalloproteinases; COX-2, cycloxygenase-2; CSF-1, colony-stimulating factor-1; SIRPα, signal-regulatory protein alpha.

**Table 3:** Treatment options with novel agents for SMM.

	Clinical Trial	Phase	Therapeutic Regimen	Patients	Follow-Up and Results
Lenalidomide-Based Treatments	QuiRedex- NCT00480363	III	RD +/− R maintenance for 2 years	119 High-risk SMM	Median FU: 10.8 years HR OS: 46% HR PFS: 73% median TTP: 9.0 years (treatment arm) vs. 2.1 years (control arm) median OS: not reached (treatment arm) vs. 7.8 years (control arm)
	ECOG E3A06-NCT01169337	III	R	182 Intermediate/high-risk SMM	Median FU: 35 months HR PFS: 72% 3 years PFS: 91% (treatment arm) vs. 66% (control group)
Proteasome Inhibitor-Based Treatments	NCT01572480	II	KRd + R maintenance	18 High-Risk SMM	Median FU: 43.3 months MRD-negative: 63% Estimated 4 years PFS: 71% Estimated 4 years OS: 100%
	GEM-CESARNCT02415413	II	KRd + high-dose Melphalan and ASCT + Rd maintenance for up to 2 years	90 High/ultra high-risk SMM	Median FU: 32 months OS: 98% PFS: 93% biochemical relapses: 5 patients ORR after induction: 98% ORR after ASCT: 98% ORR after consolidation: 100% CR: 68.6% (55% of them achieving MRD negativity)
	NCT02916771	II	IxRd + IxR mainteinance	26 (56 planned) High-risk SMM	ORR: 89% (after at least 3 cycles of treatment) CR: 19.2% No progression to active MM.
Monoclonal antibody-Based Treatments	CENTAURUS NCT02316106	II	Dara (three different treatment schedules: extended intense, extended intermediate and short dosing)	123 Intermediate/high-risk SMM	Median FU: 26 months CR: 4.9%, 9.8%, 0% (respectively, in the three treatment schedules) 2 years PFS: 89.9%, 82%, 75,3% (respectively, in the three treatment schedules)
	NCT03236428	II	Dara	28 Lower-risk SMM	PR: 53% VGPR: 20%
	NCT02960555	II	Isatuximab	24 High-risk SMM	PR: 42% VGPR:17% CR with MRD negativity: 5%
	NCT01441973	II	Elo	31	FU > 28 months Modest activity of Elo monotherapy. ORR: 10% 2-year PFS: 69%
	NCT02279394	II	EloRd + EloR	50 High-risk SMM	PR: 84% No progression to active MM.
	NCT02603887	Pilot study	Pembrolizumab	13 Intermediate/high-risk SMM	After a median of 8 cycles: 85% CR, 15% progressed to active MM, 8% MRD negativity (for up 27 months)
	NCT01484275	Pilot study	Siltuximab	85 High-risk SMM	Median FU: 29.2 months 1-year PFS: 84.5% (siltuximab) vs. 74.4% (placebo) median PFS: not reached (siltuximab) vs. 23.5 months (placebo) OS: not reached in both arms
Vaccines	NCT01718899	I/IIa	PVX410 +/− R	20 Intermediate/high-risk SMM	progressions: 3 (PVX-410-alone) vs. 1 (PVX-410 + R) median TTP: 36 weeks (PVX-410-alone) vs. not reached (PVX-410 + R)
Ibrutinib	NCT02943473	II	Ibrutinib	9 High-risk SMM	poor efficacy unfavorable risk/benefit ratio

Lenalidomide-Based Treatments for SMM.

## Data Availability

Not applicable.
